# Erotylidae (Insecta, Coleoptera) of Poland – problematic taxa, updated keys and new records

**DOI:** 10.3897/zookeys.134.1673

**Published:** 2011-10-06

**Authors:** Rafał Ruta, Paweł Jałoszyński, Paweł Sienkiewicz, Szymon Konwerski

**Affiliations:** 1 Department of Biodiversity and Evolutionary Taxonomy, Zoological Institute, Wrocław University, Przybyszewskiego 63/77, 51–148 Wrocław, Poland; 2 Museum of Natural History, Wrocław University, Sienkiewicza 21, 50–335 Wrocław, Poland; 3 Department of Entomology and Landscape Protection, Poznań University of Life Sciences, Dąbrowskiego 159, 60–594 Poznań, Poland; 4 Natural History Collections, Adam Mickiewicz University, Umultowska 89, 61–614 Poznań, Poland

**Keywords:** Coleoptera, Erotylidae, Cryptophilinae, Erotylinae, *Cryptophilus*, *Triplax carpathica*, new records, Central Europe, Poland, Białowieża Primeval Forest

## Abstract

New data concerning the occurrence of pleasing fungus beetles (Coleoptera: Erotylidae) in Poland are given, with a focus on rare and difficult to identify Central European taxa. *Cryptophilus cf. integer* (Heer) (Cryptophilinae) is reported from the Polish territory for the first time based on adult and larval specimens collected in the Wielkopolska-Kujawy Lowland. Identification problems concerning species of *Cryptophilus* introduced to Europe are discussed. *Triplax carpathica* Reitter (Erotylinae) is recorded from the Białowieża Primeval Forest, which is the first known non-Carpathian finding of this species, located in the close proximity of the Polish-Belarussian UNESCO World Heritage Site “Białowieża Forest”. Discussion of *Triplax carpathica* being conspecific with Siberian *Triplax rufiventris* Gebler is provided. New Polish localities of several other Erotylidae are reported, and an updated key to Central European species of *Triplax* is given. The *Triplax* key is supplemented with dorsal and ventral habitus images of all treated *Triplax* species. One of the rarest Central European erotyline species *Combocerus glaber* (Schaller) is recorded from xerothermic grasslands in North-West Poland.

## Introduction

The systematics of pleasing fungus beetles (Erotylidae) has undergone significant changes within the past decade, and according to the currently accepted classification (Węgrzynowicz 2007) all species hitherto known to occur in Poland belong to the subfamily Erotylinae. Four genera have been recorded from Poland: *Combocerus* Bedel, *Dacne* Latreille (both in the tribe Dacnini), *Triplax* Herbst and *Tritoma* Fabricius (tribe Tritomini) (Mazur 1983; Burakowski et al. 1986; Węgrzynowicz 2007). The larvae of *Combocerus* remain unknown, but adults occur in plant debris (Franc 2001); whereas the remaining genera develop in saproxylous fungi (Burakowski et al. 1986). Most European Erotylidae are included in the European red list of saproxylic beetles (Nieto and Alexander 2010).

Distributions of most Erotylidae in Poland are inadequately known and published faunistic data are relatively scarce. Some species have only been recorded from the southern arch of mountains or from few localities within the most species-rich (and intensively surveyed) national parks, but their true ranges may be broader. For example, *Triplax collaris* (Schaller) was previously known in Poland only in the Białowieża Primeval Forest (Burakowski et al. 1986), but it was recently found in a distant and distinctly different forest near Poznań (Jałoszyński and Węgrzynowicz 2007).

Herein, we extend the Polish checklist of pleasing fungus beetles by reporting new findings; we also give new district records of several rare Erotylidae, and discuss taxonomic problems concerning two species.

## Methods and conventions

**Depositories:** [HNHM] – Hungarian Museum of Natural History (Budapest, Hungary); [LBJK] – coll. L. Borowiec et J. Kania (Wrocław, Poland); [MIZ] – Museum and Institute of Zoology of Polish Academy of Sciences (Warsaw, Poland); [MNHW] – Museum of Natural History, Wrocław University (Wrocław, Poland); [SMNS] – Staatliches Museum für Naturkunde (Stuttgart, Germany); [PJ] – coll. P. Jałoszyński (Wrocław, Poland); [RR] – coll. R. Ruta (Wrocław Poland); [SK] – coll. S. Konwerski (Poznań, Poland).

**Specimen handling and imaging.** Dry-mounted adult specimens and an ethanol-preserved larva were used for the study; when necessary beetles were detached in warm water from mounting cards to examine ventrites. The body length was measured from the anterior margin of pronotum to the apex of elytra. Specimens showed on color plates are from MIZ, SMNS, PJ and RR. Habitus images were taken by a Nikon Coolpix 4500 camera mounted to a Nikon SMZ 1500 dissecting microscope (all species of *Triplax*) or by an Olympus C-750UZ camera equipped with a Raynox MSN-505 close-up lens (larva and adult of *Cryptophilus*). Image stacks were processed using Helicon Focus 4.62 and Photoshop 7.0 CE (*Triplax*) or Combine ZP and Corel Photo-Paint 8 (*Cryptophilus*). Scanning electron micrographs of uncoated specimens were taken by a HITACHI S-3400N scanning electron microscope at MIZ.

**Collectors:** JK – J. Kania; JMG – J. M. Gutowski; JS – J. Szypuła; JSA – J. Sawoniewicz; KK – K. Kowalczyk; LB – L. Borowiec; LBU – L. Buchholz; MW – M. Wanat; PJ – P. Jałoszyński; PS – P. Sienkiewicz; RR – R. Ruta; SK – S. Konwerski.

**Other abbreviations:** f. sec. – forest section;nat. res. – nature reserve; N.P. – national park; BL – body length; BW – maximum body width. An asterisk is used to mark first species records for particular districts of Poland (according to the division of Burakowski et al. 1973).

## Taxonomy

### Cryptophilus cf. integer

(Heer, 1841)

http://species-id.net/wiki/Cryptophilus_integer

Figs 1, 2 

#### Material examined.

 Poland*, Wielkopolska-Kujawy Lowland: Włocławek (UTM: CD63), compost heap, 1 adult and 1 larva, 26 VIII 2010, leg. PJ [PJ].

In the Palearctic Region, Cryptophilinae are represented by a single species of the East Chinese genus *Chinophagus* Lyubarsky, and the broadly distributed *Cryptophilus* Reitter with nine species (Węgrzynowicz 2007). External morphology of *Cryptophilus* superficially resembles that of many Cryptophagidae and for a long time members were placed in that family, despite historical work by Ganglbauer who reclassified Erotylidae and included *Cryptophilus* in Diphyllini, Erotylinae (Ganglbauer 1899). However, Ganglbauer’s broad concept of Erotylidae (including Cryptophagidae and Biphyllidae) was disputable and not followed by subsequent authors. *Cryptophilus* was included in Languriidae (currently Languriinae within Erotylidae) by Sen Gupta and Crowson (1971) and Lawrence (1991); in Erotylidae, Cryptophilinae by Chûjô (1969); in Erotylidae, Xenoscelinae by Węgrzynowicz (2002); and in Erotylidae, Cryptophilinae in major modern catalogues and revisions (e.g. Leschen and Buckley 2007; Węgrzynowicz 2007).

The only *Cryptophilus* species reported from many European countries, including western and southern neighbours of Poland, is *Cryptophilus integer* (Heer, 1841). The species was originally described in *Cryptophagus* Herbst, and indeed can be easily misidentified by an inexperienced coleopterist as a member of the Cryptophagidae. The latter family has never been popular among Polish entomologists and species in some genera (e.g. *Cryptophagus*) are difficult to identify. Therefore, the fact that *Cryptophilus* has been found only recently in Poland can be explained either by a possible misidentification as Cryptophagidae in institutional and private collections, or by current expansion or introduction. The major difference between cryptophagids and cryptophilines is the developement of the procoxal sockets, which are open or nearly open in Cryptophagidae and closed in Erotylidae. The larvae of *Cryptophilus* (Fig. 2) can be possibly misidentified as *Monotoma* Herbst (Monotomidae) or *Epuraea* Erichson (Nitidulidae); they all share a similar body shape and granulate or tuberculate dorsum, and occur in similar habitats (often together). Unambiguous determination of Central European specimens can be made based on structures of the head capsule, mouthparts and terminal abdominal segments. Among others, the shape of the mandibles is clearly different: in *Cryptophilus* the prostheca is very large, subtriangular, and the mandible lacks a subapical accessory tooth; in *Monotoma* the prostheca is inconspicuous and the subapical accessory tooth present, very long and slender; in *Epuraea* the prostheca is developed as an elongate brush of hairs and the accessory tooth is absent.

*Cryptophilus integer* is associated with decaying plant matter (e.g. compost heaps). The genusneeds a comprehensive revision before world species can be confidently identified. Due to this taxonomic problem the true identity of species recorded from Europe requires verification by specialists (Węgrzynowicz, pers. comm.). Therefore, although presenting the first Polish finding of *Cryptophilus* is justified, the identification of specimens must be treated as uncertain. Therefore we treat all previously published European records of *Cryptophilus* as tentatively identified pending verification by comparison of type specimens.

**Figures 1–2.**
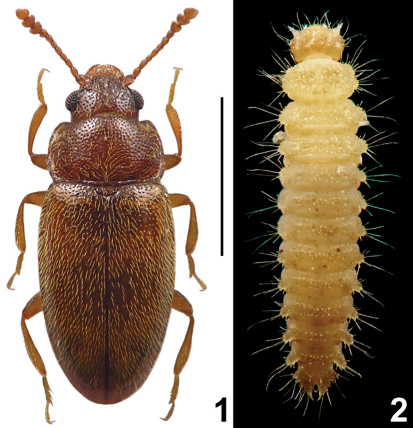
*Cryptophilus* cf. *integer* (Heer); habitus of adult **1** and larva **2** Scale bar = 1 mm.

### Triplax carpathica

Reitter, 1890

http://species-id.net/wiki/Triplax_carpathica

Figs 4 15 

#### Type material examined.

 Holotype, **Romania** (originally “Hungaria bor.”), Marmaros, Coll. Reitter [HNHM].

**New material:** Poland, Białowieża Primeval Forest*: Czerlonka vic., f. sec. 494 (FD84), 1 X 2000, 1 ex., leg. JMG [RR]; Olszanka-Myśliszcze nat. res. (FD83), 17 VIII 2000, 1 ex., leg. MW [MNHW]. **Bieszczady Mts.:** Wetlina, Muchanin Wierch (FV04), 20 VIII 1970, 6 exx. [MIZ].

Among the beetles recently collected in the Białowieża Primeval Forest, two specimens possibly belonging to *Triplax carpathica* were found. This species was described from Romanian Carpathians (Reitter 1890), and recorded in Poland only from Bieszczady Mts. (Borowiec 1984). Its occurrence outside the Carpathian Range in northeastern Poland seemed highly unlikely. To unambiguosly confirm the species identity, the holotype of *Triplax carpathica* was examined, and the Polish specimens were found to be conspecific with the type. It is plausible that *Triplax carpathica* is another species with primarily Eastern Palearctic distribution that extends westwards to the Białowieża Primeval Forest in Central Europe (see the discussion of taxonomic problems below). The rare beetles *Pytho kolwensis* Sahlberg (Pythidae) and *Xylobanellus erythropterus* (Baudi) (Lycidae) are other examples of a similar distribution. The new findings of *Triplax carpathica* confirm the important value of woodlands surrounding the protected part of the Białowieża Forest, which is one of the last and largest primeval forests in Europe. Only a part of the forest is currently included in the Białowieża National Park and a UNESCO World Heritage Site. However, adjacent unprotected areas also show a remarkable biodiversity and may be important for survival of disjunct populations of *Triplax carpathica* and other rare insects.

A taxonomic problem was encountered when characters of the holotype of *Triplax carpathica* were compared with existing descriptions and keys.Previously, the only feature reported to differentiate *Triplax carpathica* from the East Palearctic *Triplax rufiventris* Gebler, 1823 (= *Triplax amurensis* Reitter, 1879), i.e. presence/absence of femoral lines, seemed dubious at best. Iablokoff-Khnzorian (1975) in his identification key stated that *Triplax carpathica*, but not *Triplax rufiventris*, has all femoral lines present (“toutes les lignes fémorales présentes”). However, the holotype of *Triplax carpathica* exhibits no traces of femoral lines on the metaventrite. Unfortunately, we were not able to study the type specimens of *Triplax rufiventris* and *Triplax amurensis* (both presumably in the Muséum National d’Histoire Naturelle, Paris) to clarify the true status of these taxa. A comprehensive revision of Palearctic *Triplax* remains beyond the scope of the present paper. However, we note that the identity of some species and their geographic distributions remain unclear and require further study.

**Figures 3–13.**
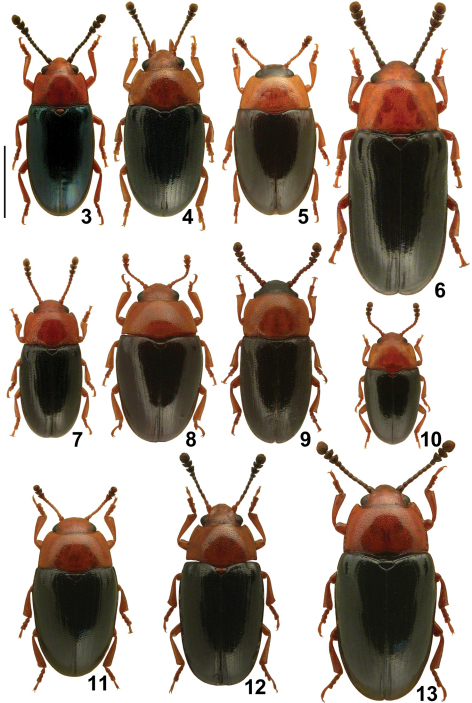
Central European species of *Triplax*, dorsal habitus: *Triplax aenea* **3** *Triplax carpathica* **4** *Triplax collaris* **5** *Triplax elongata* **6** *Triplax lacordairei* **7** *Triplax lepida* **8** *Triplax melanocephala* **9** *Triplax pygmaea* **10** *Triplax rufipes* **11** *Triplax russica* **12** and *Triplax scutellaris* **13** Scale bar = 1.0 mm.

**Figures 14–24.**
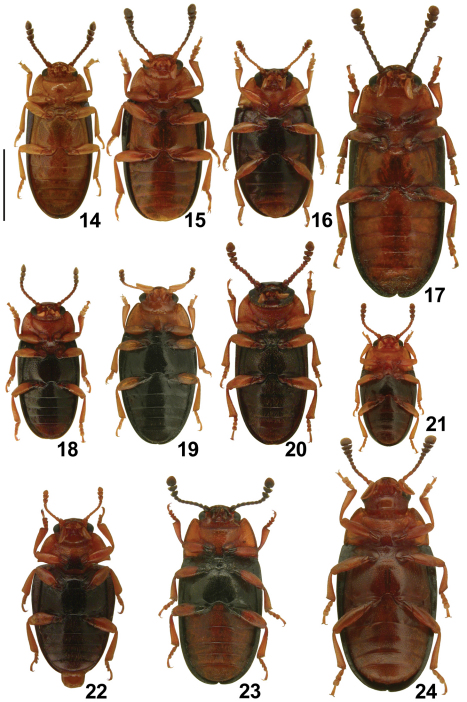
Central European species of *Triplax*, ventral habitus: *Triplax aenea* **14** *Triplax carpathica* **15** *Triplax collaris* **16** *Triplax elongata* **17** *Triplax lacordairei* **18** *Triplax lepida* **19** *Triplax melanocephala* **20** *Triplax pygmaea* **21** *Triplax rufipes* **22** *Triplax russica* **23** and *Triplax scutellaris* **24** Scale bar = 1.0 mm.

### Triplax aenea

(Schaller, 1783)

http://species-id.net/wiki/Triplax_aenea

Figs 3 14 

#### Material examined.

 **Baltic Coast:** Lubin (VV66), Wolin Is., 3 V 1991, 5 exx., leg. MW [MNHW]. Pomeranian Lake District*: Szczecin (VV71), 2 VI 2004, 2 exx. caught in sticky trap on *Aesculus hippocastanum* in city park “Ogród Dendrologiczny im. Stefana Kownasa”, leg. SK [SK], 3 VI 2004, 3 exx. under bark of a rotten trunk in city park “Park im. Fryderyka Chopina”, leg. SK [SK]; Stara Rudnica (VU45) ad Cedynia, 29 IV 2010, 1 ex. on freshly cut poplar, moist meadow, leg. PJ [PJ]. Podlasie*: Sobibór (FC80) ad Włodawa, ad Bug river (sifted), 28 VII 2001, 1 ex., leg. MW [MNHW]. **Białowieża Primeval Forest:** Białowieża (FD94), meadows near N.P. 13 VI 1983, 3 exx., leg. MW [MNHW]; Białowieża N.P. (FD94), 15–27 VI 1991, 1 ex., leg. LB [LBJK], f. sec. 398, 27 VI 1991, 1 ex., leg. MW [MNHW]. **Lower Silesia:** Wrocław-Świniary (XS37), 27 IV 1991, 4 exx., leg. LB [LBJK]. **Kraków-Wieluń Upland:** Ojcowski N.P., Chełmowa Góra (DA16), 13 VI -1 VII 2004, 1 ex., leg. LBU [RR]. Roztocze*: Bukowa Góra nat. res. (FB30), 21 IX 1987, 3 exx., 9 VI 1988, 2 exx., 12 VI 1989, 3 exx., 17 VI 1989, 1 ex., leg. LB [LBJK], 22–23 VI 1990, 2 exx., leg. JK [LBJK]. **Bieszczady Mts.:** Wetlina PGR (FV04), 20 VII 1994, 4 exx., 22 VII 1994, 2 exx., leg. LB [LBJK]; Wetlina-Jawornik (FV04), 22 VII 1968, 1 ex., leg. K. Smulikowski [MIZ].

One of the most common *Triplax* species in Poland, but previously not recorded from several districts.

### Triplax collaris

(Schaller, 1783)

http://species-id.net/wiki/Triplax_collaris

Figs 5 16 

#### Material examined.

 **Białowieża Primeval Forest:** Białowieża vic. (FD84), f. sec. 425, oak-hornbeam forest, 12 VI 1983, 1 ex., leg. MW [MNHW]; Białowieża N.P. (FD94), 15–27 VI 1991, 10 exx., leg. LB, f. sec. 399, 18 VI 1991, 1 ex., leg. JK [LBJK].

The only well documented localities of this rare species are in the Białowieża Primeval Forest and Wielkopolska-Kujawy Lowland (Jałoszyński and Węgrzynowicz, 2007).

### Triplax rufipes

(Fabricius, 1787)

http://species-id.net/wiki/Triplax_rufipes

Figs 11 22 

#### Material examined.

 Podlasie*: Białystok (FD49), Antoniuk nat. res., 11 VII 2001, 1 ex., leg. JSA [MNHW]; Białowieża (FD94), meadows near N.P. 13 VI 1983, 3 exx., leg. MW [MNHW]; Białowieża N.P. (FD94), f. sec. 368/398/399, 2 exx., leg. MW [MHNW]. Roztocze*: Bukowa Góra nat. res. (FB30), 9 VI 1988, 2 exx., 8 VI 1989, 2 exx., leg. LB [LBJK], 22–23 VI 1990, 4 exx., leg. JK et LB [LBJK]. **Świętokrzyskie Mts.:** Białe Ługi nat. res. (DB82), 8 VII 2007, 1 ex., leg. RR [RR]; Świętokrzyski N.P., Święta Katarzyna (DB93), f. sec. 147g, 12–25 VIII 2009, 1 ex., leg. LBU [RR]. Sudety Zachodnie Mts.*: Szklarska Poręba Średnia (WS33), 16–24 VII 1995, 1 ex., leg. LB [LBJK].

A rare species, recently recorded from the southern part of Poland.

### Triplax russica

(Linnaeus, 1758)

http://species-id.net/wiki/Triplax_russica

Figs 12 23 

#### Material examined.

 **Pomeranian Lakeland:** Bielinek nad Odrą (VU46), 29 IV 2010, 2 exx. on Polyporaceae fungi growing on a beach tree, leg. PJ & PS [PJ]; Bukowskie Bagno nat. res. (WU98) ad Niekursko, 30 V 2006, 1 ex., leg. RR [RR]. **Wielkopolska-Kujawy Lowland:** Biedrusko vic. (XU22), military range, 24 VI 2006, 1 ex., 20 VIII 2007, 1 ex., both in hornbeam-oak forest , leg. SK [SK]; Rogalin (XT38) ad Poznan, 17 V 2011, leg. PS [PJ]; Piła-Kalina (XU28), 9 V 1998, 1 ex., leg. RR [RR]; Skoroszów (XT50), 6 VIII 1991, 1 ex., leg. LB [LBJK]; Stawy Przemkowskie nat. res. (WT51), 20 V 2007, 1 ex., leg. LB [LBJK]; Poznań, Maltańskie lake (XU30), 14 VI 2004, 1 ex. on a sticky trap on a pine tree, leg. SK [PJ]; Puszczykowo vic. ad Poznań (XT29), 21 V 2010, 1 ex. on a fungus growing on a beech tree; 8 VI 2010, 2 exx. on a fungus growing on a beech tree, leg. PJ [PJ]; Buczyna Szprotawska (WT40), 19 V 2007, 2 exx., leg. RR [RR]. Podlasie*: Sobibór (FC80) ad Włodawa, ex *Leccinum scabrum*, 4–6 VIII 2000, 1 ex., leg. MW [MNHW]. **Lower Silesia:** Lwówek Śląski (WS46), in an oak alley, VII 2007, 1 ex., leg. RR [RR]. **Świętokrzyskie Mts.:** Św. Krzyż (EB03), 6 VII 1978, 6 exx., leg. KK [MNHW], 21 V 1992, 3 exx., leg. LB [LBJK]. **Roztocze:** Bukowa Góra nat. res. (FB30), 9 VI 1988, 2 exx., 17 VI 1989, 1 ex., leg. LB [LBJK], 22–23 VI 1990, 2 exx., leg. JK [LBJK]. **Bieszczady Mts.:** Wetlina PGR (FV04), 22 VII 1994,2 exx., leg. LB [LBJK]; Wetlina-Jawornik (FV04), 22 VII 1968, 1 ex., leg. K. Smulikowski [MIZ]; Wetlina, Muchanin Wierch (FV04), 20 VIII 1970, 6 exx. [MIZ].

The most common species of *Triplax* in Poland.

### Triplax scutellaris

Charpentier, 1825

http://species-id.net/wiki/Triplax_scutellaris

Figs 13 24 

#### Material examined.

 **Białowieża Primeval Forest:** Białowieża N.P., 4 VIII 1992, 1 ex., leg. JS [LBJK]. **Bieszczady Mts.:** Wetlina PGR (FV04), 20 VII 1994, 3 exx., 22 VII 1994, 2 exx., 24 VII 1994, 9 exx., 27 VII 1994, 2 exx., leg. LB [LBJK]; Wetlina-Jawornik (FV04), 22 VII 1968, 3 exx., leg. K. Smulikowski [MIZ].

This species has been rarely collected in Poland. However, the new findings demonstrate that *Triplax scutellaris* is not uncommon in the Bieszczady Mts.

### Combocerus glaber

(Schaller, 1783)

http://species-id.net/wiki/Combocerus_glaber

#### Material examined.

 Wielkopolska-Kujawy Lowland*: Laski (VU71), xerothermic grassland *Potentillo*-*Stipetum* with *Stipa joannis* Čelak. in a deep ravine surrounded by fields, 4 VI 2009, 1 ex., leg. PS [SK], 28 V 2010, 1 ex., leg. PS [PJ]; Laski (VU71), xerothermic grassland *Adonido*-*Brachypodietum* in a deep ravine surrounded by fields, 28 V 2010, 1 ex., leg. PS [PJ].

This species is rare in Poland, although it has been recorded from scattered localities (Burakowski et al. 1986), and was recently recorded in Poland in the Białowieża Primeval Forest (Byk et al. 2006).

### Tritoma subbasalis

Reitter, 1896

http://species-id.net/wiki/Tritoma_subbasalis

#### Material examined.

 Podlasie*: Szostaki (EE90) ad Burzyn, 17 VI 2010, 3 exx., leg. RR [RR, SK]. Lublin Upland*: Poleski N.P., Łukie Lake vic. (FB49), 22 V 2004, 2 exx., leg. RR [RR].

This rare species is restricted to the eastern part of Poland.

### Dacne rufifrons

(Fabricius, 1775)

http://species-id.net/wiki/Dacne_rufifrons

#### Material examined.

 **Bieszczady Mts.:** Wetlina State Agricultural Farm (FV04), 27 VII 1994, 2 exx., leg. JK [LBJK].

A rare species, known from scattered localities in various regions of Poland.

##### Updated key to Triplaxspecies of Central Europe

*Triplax carpathica* was not included in any of the previously published identification keys to Central European Erotylidae (Vogt 1967, Mazur 1983). Therefore, an updated key is presented below, comprising all Central European *Triplax* species (Figs 3–24). Species known from adjacent areas and which may occur in Poland are denoted with an asterisk.

**Table:** 

1	Basal margin of elytra crenulate (Fig. 25), often forming a distinct ridge	2
–	Basal margin of elytra smooth, without crenulation (Fig. 26)	9
2	Head black	3
–	Head yellowish-red	4
3	Body elongate oval, distinctly rounded; BL 3.3–4.0 mm	*Triplax melanocephala* (Latreille)*
–	Body broadly oval, sides distinctly rounded; BL 3.3–4.0 mm	*Triplax collaris* (Schaller)
4	Entire venter yellowish-red	5
–	Meso- and metaventrite black	6
5	Elytra metallic bluish or greenish; BL 3.6–4.4 mm; sides of body rounded	*Triplax aenea* (Schaller)
–	Elytra black, without metallic hue; BL 6.0–6.5 mm; sides of body subparallel	*Triplax elongata* Lacordaire
6	Abdominal sternites yellowish-red; BL 5.1–6.7 mm	*Triplax russica* (Linnaeus)
–	Abdominal sternites entirely or largely black	7
7	Body oval, sides rounded; BL 3.9–4.1 mm	*Triplax rufipes* (Fabricius)
–	Body elongated, sides subparallel	8
8	Antennomere III much longer than II; BL 3.0–4.2 mm	*Triplax lacordairei* Crotch*
–	Antennomere III slightly longer than II; BL 2.0–3.0 mm	*Triplax pygmaea* Kraatz*
9	Meso-, metaventrite and abdominal sternites black; body stout (BL/BW 1.7–1.8); BL 3.3–5.0 mm	*Triplax lepida* (Faldermann)
–	Entire venter yellowish-red, body larger and more oblong (BL/BW 1.9–2.0)	10
10	Scutellum yellowish-red, elytral punctures shallow; BL 4.2–4.9 (n=16, mean 4.6)	*Triplax scutellaris* Charpentier
–	Scutellum always black, eelytral punctures deep; BL 3.9–4.5 (n=9, mean 4.1)	*Triplax carpathica* Reitter

**Figures 25–26.**
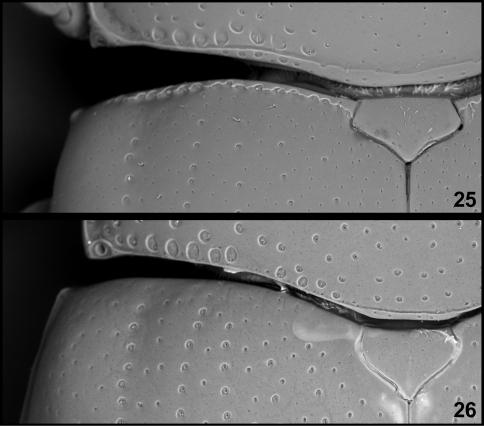
*Triplax*, basal part of elytra (SEM micrographs); *Triplax russica* **25** and *Triplax scutellaris* **26**.

## Supplementary Material

XML Treatment for Cryptophilus cf. integer

XML Treatment for Triplax carpathica

XML Treatment for Triplax aenea

XML Treatment for Triplax collaris

XML Treatment for Triplax rufipes

XML Treatment for Triplax russica

XML Treatment for Triplax scutellaris

XML Treatment for Combocerus glaber

XML Treatment for Tritoma subbasalis

XML Treatment for Dacne rufifrons
